# Mismatch Negativity in Children with Deficits in Auditory Abilities

**DOI:** 10.1055/s-0044-1785458

**Published:** 2024-05-25

**Authors:** Julia Dalcin Pinto, Déborah Aurélio Temp, Laís Ferreira, Amália El Hatal de Souza, Michele Vargas Garcia, Adriana Neves de Andrade, Eliara Pinto Vieira Biaggio

**Affiliations:** 1 Department of Speech Therapy, Centro de Ciências da Saúde, Universidade Federal de Santa Maria (UFSM), Santa Maria, RS, Brazil; 2 Department of Speech Therapy, Escola Paulista de Medicina, Universidade Federal de São Paulo, São Paulo, SP, Brazil

**Keywords:** auditory cortex, central auditory processing, central auditory processing disorder, child, auditory evoked potentials

## Abstract

**Introduction** Mismatch negativity (MMN) represents a negative component of event-related potentials, which is mentioned by guidelines as an important tool to provide measurable data regarding the functionality of the auditory system in acoustic processing. However, the literature still lacks reliable data that can support the clinical use of this potential in the complementary diagnosis of central auditory processing (CAP) disorder (CAPD).

**Objectives** To analyze whether MMN assessment might be associated with the CAP behavioral test battery, as well as to assess the effects of auditory ability deficits on MMN responses in the pediatric population.

**Methods** In total, 45 age-matched children participated in the study. They were submitted to the CAP behavior assessment and to MMN. The children were tested with a combination of speech contrast consisting of acoustic syllables [da] versus [ta], governed by the oddball paradigm.

**Results** Mismatch negativity did not show a direct association with a single test but with the combination of the four tests used as a behavioral test battery to identify CAPD. The results also indicated that the auditory ability deficits influenced the measurement of MMN latency ( *p* = 0.003*), but not the amplitude ( *p* = 0.857) or the area ( *p* = 0.577).

**Conclusion** Mismatch negativity was shown to be statistically associated with the battery of tests used to identify deficits in auditory abilities in the studied sample rather than with a single behavioral test. The deficits in auditory abilities were observed in the MMN latency. Mismatch negativity can be used to assess children with CAPD.

## Introduction

Central auditory processing (CAP) refers to several neural mechanisms which, combined, are responsible for interpreting auditory information, 1 that is, a set of auditory abilities that provide meaning to the acoustic stimuli. These abilities demonstrate the make it possible for the central auditory nervous system (CANS) to locate and lateralize acoustic phenomena, auditorily distinguish differences in sounds, recognize and order temporal aspects, as well as integrate acoustic signals with different types of degraded speech and competing messages. 2

Deficits in at least one auditory ability in this perception process characterize CAP disorder (CAPD), 3 which may affect populations of various age groups in different proportions. In the pediatric population, ∼ 3% to 7% of school-age children may present CAPD. 4 This disorder is diagnosed through a series of behavioral tests, electrophysiological measurements, and complementary tests, such as speech and language assessments. 1

The behavioral assessment method consists of associating various tests that evaluate the mechanisms related to dichotic listening, temporal processing, low-redundancy monaural speech perception, location, lateralization, and binaural integration. 1 2 This set of tests provides evidence of functional alterations pervading the process of perceiving and interpreting auditory information. Nevertheless, innumerable variables may influence the results of this method, such as the attention 5 and motivation of the subject. 6

In this light, electrophysiological methods have been proposed as an alternative to identify CAPD without relying solely on the participation of the child in the assessment, as we know reliable responses are often difficult to be obtained from children and may influence the accuracy of the diagnosis. 1

Among the electrophysiological tests used to complement the diagnosis of CAPD, mismatch negativity (MMN) stands out, representing a negative component of event-related potentials (ERPs). This component enables the evaluation of the cortical region and adjacent areas, such as the thalamus and hippocampus, which are essential to auditory perception and discrimination. 7 Mismatch negativity is elicited when a violation of the regularity of acoustic stimuli is detected, reflecting involuntary attention and the ability of the brain to distinguish different stimuli stored in memory. 7

Even though, within the scientific community, MMN is referred to as a relevant tool to complement the diagnosis of CAPD, 1 little is known about the applicability of this potential to assess the neurophysiological bases that permeate auditory information processing in this disorder. 8 9 10 11

The use of verbal stimuli in the MMN assessment is an effective alternative to bring the electrophysiological assessment nearer to the conditions of the behavioral testing of CAPD. 2 Also, it is an important feature to use with children, as the use of verbal stimuli may somehow mimic the conventional day to day settings to which the child is exposed. Nonetheless, few studies 8 9 11 have sought to elicit MMN with verbal stimuli in subjects with CAPD, furthermore, with diverging results.

Therefore, the aim of the present study was to analyze whether the MMN assessment might be associated with the CAP behavioral test battery, as well as to assess the effects of auditory ability deficits on MMN responses in the pediatric population.

## Methods

### Ethics

The present is an observational, cross-sectional, and quantitative study, approved by the institutional Ethics in Research Committee (under number 81117517.0.0000.5346 and opinion 2.538.043). The legal guardians of the children that composed the sample were informed about the procedures, and those who agreed to participate signed a written informed consent term.

### Participants

The present is a study of a convenience sample, comprising 45 children of both genders, whose ages ranged from 5 to 11 years and 11 months. Data collection occurred from August to December 2018 in a school clinic. The children were recruited from both the school clinic of the institution of the study and from local public schools.

### Inclusion and Exclusion Criteria

The inclusion criteria considered for the study and control groups were the following: all children must have presented normal hearing thresholds 12 and normal auditory brainstem responses (ABRs) 13 elicited with click stimuli. The ABR was recorded prior to MMN to ensure that the participants presented normal auditory pathway synchrony. Also, all children had to present adequate performance in the noninstrumental language assessment, which was performed with the purpose of ruling out any alterations pervading the aspects of syntax, vocabulary, pragmatics, and semantics that could influence the accuracy of the data. This assessment was undertaken through the presentation of a logical sequence of images, whose events the child was supposed to correctly order and spontaneously describe to analyze the speech aspects previously mentioned. The outcomes of this screening were considered satisfactory if the child did not present any phonological errors, articulatory blocks, or reductions in vocabulary. This type of rapid screening ensured that language deficits were not influencing the results obtained in the study.

The exclusion criteria were: being bilingual and/or exposed to musical education; having a previous diagnosis of a hyperactive and/or attention disorder; and no apparent neurological or psychiatric dysfunction. The children who, for some reason, did not complete the proposed behavioral and/or electrophysiological assessments were also excluded from the study.

### Behavioral Measures

The children were divided into two groups according to the results obtained in the scale of auditory behavior (SAB) 14 and in the CAP behavioral assessment. The SAB is a 12-question form answered by the children's legal guardians regarding the daily activities as well as the auditory behavior of the child, with the objective of indicating a possible risk of developing CAPD. The SAB was used as a complementary screening instrument for the identification of children at risk of developing auditory deficits; scores ≥ 46 points indicate normality according to the scale, whereas lower scores suggest risk of developing CAPD. 14

The behavioral CAP assessment comprised the dichotic digits test (DDT), 15 the random gap detection test (RGDT), 16 the speech perception in noise (SPN) test, 15 and the masking-level difference (MLD) test. 17 These tests were selected considering the age range of the sample, based on Brazilian norms and international guidelines. 1 2

The outcomes of the CAP assessment were classified as normal if the children presented responses in all four tests previously mentioned within the reference range established by each protocol according to the age of the children, or abnormal if the children did not respond within the reference range for their age for at least one of the tests.

### Sample Groups

Considering the eligibility criteria and the results from the CAP behavioral assessments, the participants were grouped as follows:

The group of children with typical development/control group (CG) was composed of 23 children, 10 male and 13 female subjects, aged between 5 and 11 (mean: 8.4; standard deviation [SD]: 1.65; minimum [min]: 5.7; maximum [max]: 11.0) years. All of them presented adequate performance in the CAP behavioral assessment and adequate scoring in the SAB (mean: 52.5 points). On the other hand, the group of children with auditory ability deficits/study group (SG) comprised 22 children, 11 male and 11 female subjects, in a similar age range (mean: 7.4; SD: 1.21; min: 5,5; max: 10.2 years), who presented poor performance in at least one of the tests used to assess the CAP. They also presented altered SAB scoring (mean: 41 points).

The children were not subdivided into different groups according to their different ages, as this would harm the statistical analysis and, therefore, the aim of the study, although all responses, both behavioral and electrophysiological, were analyzed according to the reference parameters for each specific age.

### Electrophysiological Measurement

Subsequently, all children were subjected to MMN recording through the Smart EP module of the Intelligent Hearing Systems (IHS, Miami, FL, United States) equipment with two channels. To improve the adhesion of the electrodes, the skin of the child was cleaned with abrasive paste. Disposable surface electrodes were then positioned in Fz, Fpz, M1, and M2, with the impedance maintained at 1 to 3 Kohms. The registers that exceeded 10% of artifacts due to environmental conditions or to the state of the children during testing were excluded from the analysis.

During testing, the children were accommodated in a comfortable armchair in a quiet room and were instructed to watch an age-appropriate movie that was playing at a low volume on a computer in front of them. They were also instructed to ignore the auditory stimuli and pay attention solely to the movie.

The children were tested with a combination of speech contrast consisting of acoustic syllables [da] versus [ta], the frequent (standard stimulus) and the rare (deviant stimulus) respectively, which were presented in a counterbalanced order.

To elicit MMN deflection, the stimuli were presented binaurally via insert earphones at 60 dBnHL, governed by a classic oddball paradigm, that is, 80% of the stimuli presented were frequent [da] and 20% were rare stimuli [ta]. A total of 750 stimuli were presented to obtain at least 150 repetitions of the rare stimuli at a rate of 1.9 stimuli per second with alternate polarity. A 1.0-KHz low-pass filter and a 30-Hz high-pass filter were used, with a recording window of 50 ms prior to the stimulus (prestimulation) and of 510 ms posterior to the stimulus. The interstimulus interval (ISI) and the duration adopted for the stimuli were of 320 ms/206.275 (μs) for [da] and of 306 ms/220.350 (μs) for [ta] respectively.

The MMN component was considered the most prominent negativity following the N1 component, 18 with a minimum amplitude value of at least -0.3 µV. Thus, the amplitude values were collected from the greatest negative point up to the prestimulation line, 19 excluding the participation of the N1 component. Values concerning the MMN area were obtained automatically by the equipment based on the amplitude and latency values previously marked.

### Statistical Analysis

The Shapiro-Wilk test was conducted to determine the distribution of data of each group. Cross-tabulation and association analyses were performed using the Chi-squared test. The Student *t* -distribution parametric test was used to compare data between both groups. The confidence intervals (CIs) were calculated with 95% statistical reliability ( *p* < 0.05).

It is important to note that no statistically significant difference was observed between the right and left ears for the analysis of the latency ( *p* = 0.752; and 0.700 respectively), amplitude ( *p* = 0.968; and 0.847 respectively), and area ( *p* = 0.499; and 0.962 respectively); therefore, the average values of both ears were considered for the statistical analysis.

## Results

Clear MMN responses were obtained in all children from the CG, while 86.3% (19) of the children in the SG elicited MMN for verbal stimuli. Thus, those who did not elicit MMN waveforms were excluded from the initial statistical analysis due to the absence of responses.

**Fig. 1:**
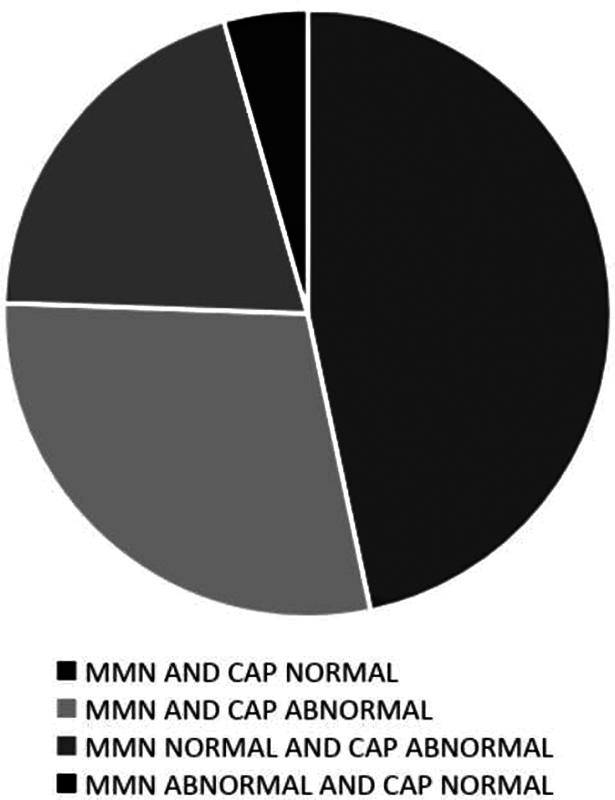
Probability relationship among the altered mismatch negativity waveforms and the data from the central auditory processing behavioral assessment ( *n* = 16).

The association analysis between the MMN assessment and the CAP behavioral battery of tests indicated that the MMN does not seem to directly associate with any individual test used to assess the auditory abilities (DDT, *p* = 0.303; RGDT, *p* = 0.474; SPN, *p* = 0.350; and MLD, *p* = 1.000). Nevertheless, a statistically significant association was obtained when analyzing the MMN assessment and the outcomes (normal or abnormal) in the entire battery of the four tests combined, that is, DDT, RGDT, SPN, and MLD ( Fig. 1 ).

**Fig. 2:**
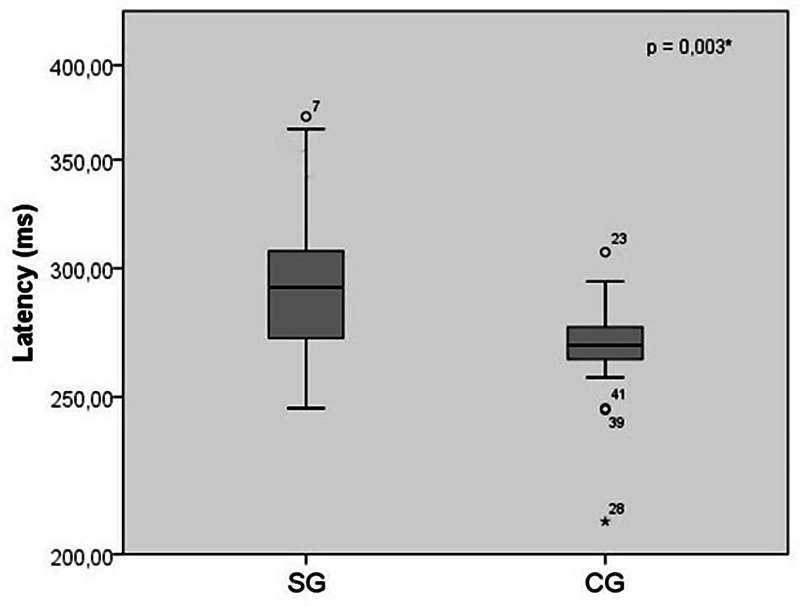
A comparison of the mismatch negativity latency values (in ms) between children with central auditory processing disorder and children with typical development ( *n* = 42). A *t* -test showed a statistically significant difference between the study group and the control group.

In the comparison analysis between the average latency values, a statistically significant difference was observed ( *p* = 0.003*) between the 2 groups studied ( Fig. 2 ). Prolonged latency values were verified for the SG.

**Fig. 3:**
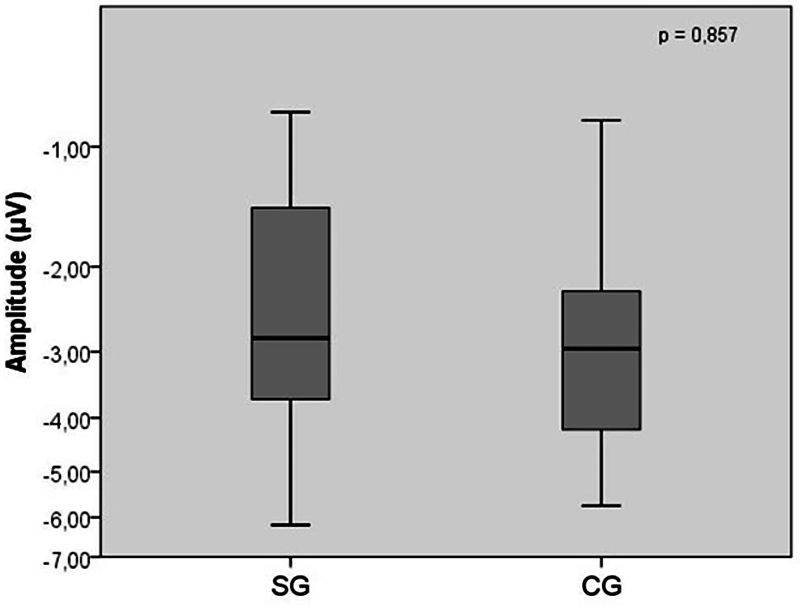
Comparison of mismatch negativity amplitude values (in μV) among children with central auditory processing disorder and children with typical development ( *n* = 42). No statistically significant difference was obtained through the *t* -test analysis between the study group and the control group.

**Fig. 4:**
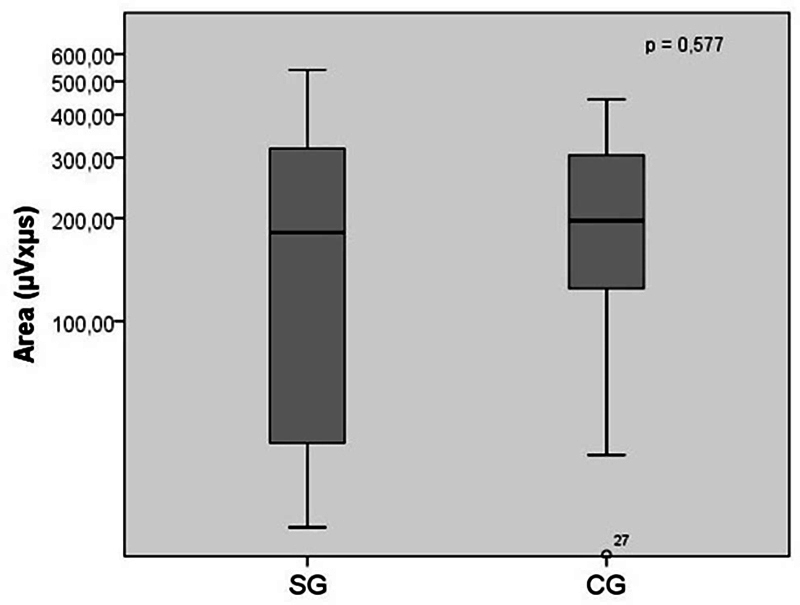
Mismatch negativity area values (in μVxms) comparison among children with central auditory processing disorder and children with typical development ( *n* = 42). No statistically significant difference was obtained through the *t* -test analysis between the study group and the control group.

*T* -tests were used to explore the statistical relationship among the average amplitude and area values between the CG and SG, and no statistically significant difference was observed between the groups in the analysis of both variables respectively ( *p* = 0.857; ( Fig. 3 ; and *p* = 0.577; Fig. 4 ).

## Discussion

Overall, the findings of the present study reveal that deficits in auditory abilities influence the MMN latency responses, suggesting that children with suspected auditory disorders may present preattentional difficulties in the discrimination of acoustic contrasts. The MMN assessment did not relate to an individual CAP test; however, a remarkable association was obtained for the analysis performed between MMN and the entire battery of behavioral tests. Therefore, this data implies that the MMN may accurately capture deficits in the central auditory pathway that end up impairing the CAP, also expressing the possibility of clinically using this potential to complement the diagnosis of CAPD.

Regarding the occurrence of MMN in the studied groups, all children in the CG elicited MMN deflection. Previous data acquired with a similar sample had also shown similar findings in the pediatric population. 10 20 21 22 However, the MMN was not elicited in all children in the SG. The absence of MMN responses already suggests a rather substantial deficit in auditory abilities; however, these were extracted from a further analysis, as there was no available value for the variables of latency, amplitude, and area to submit to the statistical handling of the data, which we discuss in detail below.

To date, few studies 7 10 18 have tried to examine the correlation and/or association between electrophysiological measures and behavioral tests to provide reliable information regarding auditory abilities and how they are reflected in both assessments. A valid approach to address this matter would provide insights into the neural correlates of the psychoacoustics behavioral tests by performing an association analysis between both assessments. 23

On this topic, the MMN did not seem to be directly associated with a behavioral test itself, but with the CAP behavioral assessment as a whole, that is, the combination of the four tests used. This result cannot be unexpected, as we know each test assesses one specific auditory ability, 15 16 17 whereas the MMN requires not only one specific ability, but rather a combination of several abilities, to account for auditory perception and discrimination, responsible for evoking the MMN responses. 7

Auditory processing is a complex mechanism that involves several auditory abilities; 1 2 therefore, no independent ability could account for an auditory processing deficit. Rather, problems with multiple abilities could indeed explain the deficits commonly observed in children with this type of complaint. In this light, these findings are consistent with data found in the literature, which emphasize the importance of establishing a test battery long enough to sufficiently provide data to close a diagnosis. 1 2

In addition to exploring the association between the MMN assessment and the behavioral test battery, we also aimed to assess the effects of auditory ability deficits on the MMN responses to examine whether the MMN could indeed be a valuable tool to contribute to the diagnosis of auditory disorders in the clinical practice.

Delayed MMN latency responses, that is, the time that the rare stimulus takes to be distinguished from the frequent one, 24 were observed in the SG ( Fig. 2 ). In previous studies performed with children, MMN deflection occurred at between 150 ms and 350 ms, with greater latency in registries elicited with verbal stimuli, as it happens with adults. 25 26 Thus, the data of the present study suggest that children with impaired auditory abilities may present cortical-level setbacks that could impair auditory discrimination when it comes to stimuli with little contrast. In line with this data, another study 10 has also shown that children with auditory processing complaints present higher latency values when compared with their typically developing peers.

It is evident that cognitive and linguistic aspects may significantly influence the MMN responses. 27 28 To obtain reliable data, a noninstrumental speech and language assessment was conducted for both groups to minimize within-subject variables and exclude any influence of language and cognitive alterations. This methodological care strengthens our data; thus, we infer that the delays verified in MMN latency values may be mostly related to auditory issues. Previous data 8 10 29 indicate that subjects with impaired auditory processing present alterations in MMN assessment, as was also observed in the present study. In general, these alterations are centered on the latency measure, also in accordance with the data presented here.

However, although a body of evidence suggests the influence of auditory ability deficits in the MMN assessment, the relationship between both is not yet a consensus in the scientific literature. Researchers have not observed significant differences in the MMN waveforms in children with auditory processing complaints. 9 11 Researchers who used nonverbal stimuli support a theory that the stimulus selection to elicit MMN may have influenced the findings. 9 Nevertheless, authors 11 who used verbal acoustic stimuli did not find such a statistical difference between children with and without CAPD either.

The sample sizes of the various studies mentioned here may have an impact on the lack of converging data regarding the occurrence of MMN in children with auditory ability deficits. Studies with larger samples observed differences between the groups, which did not occur in studies 9 with smaller samples (< 15 subjects).

In addition, short- and long-term memory also play an important role in eliciting the MMN response. 28 30 Generally, memory complaints are a common marker in the clinical history of patients with impaired auditory processing, thus underscoring the actual possibility of registering an altered MMN in children with these deficits.

In contrast, the influence of auditory ability deficits on both the amplitude and area measures was not evidenced ( Figs. 3 4 ). A great divergence regarding the amplitude measure, that is, the ability of the auditory system to distinguish the frequent stimulus from the rare one, 31 is found in the scientific literature, due precisely to the different protocols employed in MMN recording. The area is calculated automatically from the latency versus amplitude values by the equipment used in the present study. This analysis of this measure is quite uncommon in the clinical practice, since only one device has this resource available. However, it may be considered an additional analysis option to measure the functionality of the neural circuit involved in auditory discrimination at higher auditory pathway levels. 19

To the best of our knowledge, the present is the first study to show an analysis of the MMN area measure among children with deficits in auditory abilities. We infer that the results expressed in the present study are due, possibly, to the fact that children experiencing such deficits might present a more similar neural recruitment ability to elicit the discrimination task, although they require longer to activate this circuit and distinguish the acoustic contrasts than their typically developing peers. We hypothesize that, as no solid diagnosis of a major disorder such as CAPD can be made considering the age of the children recruited, these deficits evidenced in the auditory abilities may be linked to delayed maturation of the auditory pathway.

Obviously, modifications in latency can indicate more than just simply an auditory pathway maturation deficit. As discussed, language, cognition, and memory play important roles in this type of response. However, by performing a language screening, we tried to minimize these influences to focus on auditory processing.

Another aspect to be considered when analyzing these data is that MMN was evoked binaurally as a way to mitigate the influence of attention in the electrophysiological responses, which was important considering our sample. Although several studies 8 18 20 have also elicited MMN binaurally, as in the present study, we must also consider the possibility of asymmetry in the response between the ears that could be better captured in a monaural assessment of the separate ears. As a possible limitation of the present study, further research on the topic and with this differentiating aspect in acquisition parameters should be designed to clarify this aspect.

Taken together, the results of the present study indicate that the MMN may provide important information on the neural substrates responsible for the auditory outcomes visualized in children with auditory ability deficits. This is particularly important for the early diagnosis of toddlers whose auditory processing tests still lack normative reference values, as well as of hard-to-assess populations, such as children with neurological deficits, syndromes, and other pathologies alike. However, we emphasize that all children with auditory processing complaints must be submitted to behavioral testing as soon as possible if normative values on the tests are available, as was the case in the present study.

## Conclusion

Mismatch negativity did not appear to be associated with one single test of CAP, but a great association was verified when analyzing MMN responses with the final outcome on the CAP behavioral assessment, that is, normal and abnormal. The auditory ability deficits influenced the MMN latency responses, suggesting that children with poor performance on the CAP behavioral tests require a longer period to discriminate the acoustic contrasts. Jointly, these data suggest that the MMN can be used to complement the diagnosis of CAPD.
